# Exploring the Application of the FOX Model to Foster Pro-Environmental Behaviours in Smart Environments †

**DOI:** 10.3390/s20164576

**Published:** 2020-08-14

**Authors:** Ane Irizar-Arrieta, Diego Casado-Mansilla, Aiur Retegi, Matthias Laschke, Diego López-de-Ipiña

**Affiliations:** 1 DeustoTech, University of Deusto, Avda. Universidades 24, 48007 Bilbao, Spain; dcasado@deusto.es (D.C.-M.); dipina@deusto.es (D.L.-d.-I.); 2 Engineering Faculty, University of Deusto, Avda. Universidades 24, 48007 Bilbao, Spain; aiur.retegi@deusto.es; 3 Ubiquitous Design, Universität Siegen, 15, 57072 Kohlbettstraße Siegen, NRW, Germany; matthias.laschke@uni-siegen.de

**Keywords:** sustainable behaviour change, human–computer interaction, design for sustainable behaviour, user modelling, behavioural theories, smart environments

## Abstract

The heterogeneity and dynamism of people make addressing user diversity and its categorisation critical factors, which should be carefully considered when developing pro-environmental strategies and interventions. Nevertheless, the complexities of individuals complicates the creation of modelling and classification systems. The aforementioned issue opens a research opportunity, which should be tackled to improve the development of human-centric systems and processes. Throughout the present piece of research, our objective is to bridge that gap by extracting knowledge and insights relating to how to address user diversity when designing technologies considering sustainable behaviour. For this, we explore the possibilities of the FOX model—an early meta-model to approach the diversity of individuals when addressing pro-environmental behaviour—to classify and understand individuals while taking their heterogeneity into account. After introducing the model, a qualitative survey of eight experts is conducted. From this study, relevant findings are analysed and exposed. Taking into account the gathered knowledge, three user profiles are developed, based on the dimensions proposed by the model. Furthermore, scenarios are created for each profile, presenting three case studies where different application modes of the model are described (personalised interventions, prediction and forecasting, and individual and collective interventions). Finally, the extracted findings are analysed, discussing the main issues related to the development of pro-environmental technologies and systems.

## 1. Introduction

Due to the increasing relevance of sustainability, environmental impact has become a critically relevant issue in the development of technologies, particularly in the field of smart environments and cities [1]. In this context, the present paper offers a continuation of a previous conference paper [2], aimed at addressing the human factor in the context of sustainable person-centric technological systems. Following the approach of Mankoff et al. [3], sustainability can be described through two main conceptualisations: (1) sustainability in design, which is related to the optimisation of the materials and processes of the associated hardware and software; and (2) sustainability through design, which refers to influencing sustainable lifestyles and the decisions of individuals. In this line, Sustainable Human–Computer Interaction (SHCI) [4] aims to enhance the environmental impact of technologies addressing the human factor. Hence, SHCI considers the relations and interactions of the individual with digital/physical systems or technologies to improve sustainability [4]. Therefore, user interactions and behaviours are analysed to (1) better understand how to face the challenges derived from the human factor; and (2) enhance the management of technological devices, systems, and processes, in order to minimise their impacts on the environment. This is a paramount factor in human-centred intelligent environments [5], where actions are usually recognised to adapt interventions and to offer feedback related to the characteristics and needs of people. Therefore, it seems necessary to assume the complexity of the individual and to understand the different issues that may influence his/her behaviours and actions. [6] In essence, the study of the diversity of individuals emerges as a relevant requirement. In this context, the fact that people are different from each other may be critical. Nevertheless, individuals themselves also differ, depending on the context and due to many various factors (e.g., an individual with the habit of always taking the stairs, instead of the lift, may change this behaviour due to a temporary injury). As Hekler et al. stated, to influence sustainability in people, behavioural theories must be taken into account [7]. Therefore, heterogeneity may be studied as a flexible and dynamic attribute to face the contextual barriers and uncertainties that could influence individual actions and behaviours. For that, as mentioned previously, this paper follows a previous research piece [2], in which the FOX model (an early meta-model to face heterogeneity understanding the behavioural constructs and dimensions) was introduced and explained. This model was created after discovering the need for implementing more flexible and multidimensional user characterisations [8,9]. In the present paper, this research work is continued, exploring the potential applications of the model and applying it to the development of user profiles and scenarios.

### 1.1. Applied Research Methods

**Figure 1:**
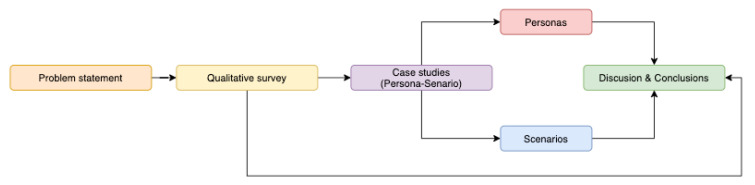
The steps of the research methodology followed in the present work.

In the following, the research approach followed in this work is explained, together with the tools and specific methodologies that are applied in this work. Figure 1 exposes the workflow and the different stages of the research process followed in this paper. The phases of the present work were followed in a linear and sequential way. However, in order to extract more information and to refine the results and insights, more iterations may be needed. This will be addressed in future research work, in order to improve and validate the findings exposed in the present paper.

Problem statement. The first step of the process starts with the definition of the problem and the research question. As the main objective is to gain knowledge on the potential applications of the FOX model, the research question arose as: How can the FOX model be applied? The complementary objective of this paper is based on gaining understanding on other relevant implications of the FOX model (i.e., improvements, shortcomings, application context, and so on).

Experimental design: qualitative survey and case studies. In order to answer the research question and to reach complementary objectives, the experimental design was centred on the analysis of the FOX model and its implications, in relation to the diversity of individuals. For that purpose, qualitative tools were selected, in order to gather rich insights and other promising information. Following the ideas exposed in a previous work [10], where the relevance of online survey tools was explained, these kinds of tools promote a user centred approach. Besides, although ethical considerations must be taken into account [10], this methodology offers a flexible and affordable method for data gathering. In addition, the importance of online surveys was already exposed by Evans and Mathur [11], providing information for approaching practical implications. The objectives of the qualitative survey were: (1) to test if the model is easily comprehensible by researchers and practitioners; (2) to discover if the FOX model is applicable in the context of smart environments; (3) to understand how and why it can be applied in those elicited contexts; and (4) to extract additional improvements and insights. This study was conducted with experts in the computer science field, and as all of the participants were already familiar with the model, it was decided to implement an online survey as a method for qualitative data gathering, following the ideas exposed by the previously mentioned research works. As the participants were used to working with the Google Suite, this survey was conducted through an online Google form, in order to provide an easy and affordable means for information collection.

Aiming at exploring how the model can be applied and to better understand the target users in their context, three qualitative case studies were developed. Following the approach of Schwandt and Gates, there is no single definition of “case study” when trying to understand the behavioural implications of individuals [12]. Besides, the authors ensured that there were considerable variations across disciplines. Taking this into account, in this paper, the definition of Schwandt and Gates is used; that is, understanding a case study as a detailed description and analysis of any specific issue, being a method widely used to perform qualitative research [12].

For the development, the Persona-Scenario method [13] was applied. This procedure integrates the Persona method with scenario building. Persona [14,15] is a common methodology in the field of HCI, which is intended to define user archetypes. It aims at envisaging a real individual, in order to gain an understanding of target users. It provides an idealisation of any given user profile, including all the relevant elements to understand the main attributes, lifestyle, emotions, feelings, and other relevant information. This tool is useful to define and visualise individuals, in relation to any given context. A recent study explored the utilisation of this tool to increase the creativity in ideation processes [16]. Hence, by developing Personas, the profile of an individual can be understood and envisaged in more depth. This is in line with the approach followed by He et al. [17] when describing the application of the transtheoretical model [18], a behavioural model to foster pro-environmentalism, and with the approach of Coskun et al. [19] when exploring user diversity. In addition, the description of the preferences and other relevant insights helps researchers and practitioners to understand better how any given intervention would be received by the potential users. The creation of scenarios is the next step of the Persona-Scenario method, which involves the exploration of the performance of the Personas in any specific context and/or system. This tool helps to envisage how any system or framework, in the context of the Personas, would work and to identify potential design problems in early stages. To better understand this behaviour, the scenarios are built using storytelling techniques, following the findings of Madsen and Nielsen [20,21]. For this, the Personas and scenariosare described as short histories, in order to better envisage the insights and implications emerging from the Personas and their interactions with the context and the system.

The paper is structured as follows: Section 2 analyses the state-of-the-art by describing the most relevant theoretical frameworks for understanding behavioural processes. Other approaches that are relevant to addressing the user diversity for sustainable behaviour are also exposed. In Section 3, the FOX model is briefly introduced to contextualise and frame the involved dimensions. In Section 4, a qualitative study to explore the potential applications of the model is exposed. Taking into account the extracted insights, three user profiles and their use contexts are described, applying the FOX model to frame them. The objective is to understand how a potential user would behave in three stereotypical contexts, according to the model. In Section 6, the results and findings are discussed, exposing relevant insights. Finally, the conclusions extracted from the research work are summarised and future research lines defined.

## 2. Background

In this section, the state-of-the-art is described, in order to analyse the most relevant works related to the research presented in this paper. Different approaches to cope with the decision-making process have been examined in the existing literature. In fact, Coskun et al. conducted an extensive review on the design of sustainability and behaviour change [22]. A recent work overviewed the main studies in relation to behavioural technologies [23], highlighting the need to address the heterogeneity of individuals when developing pro-environmental strategies in the conclusions. In order to frame the theoretical context, first, we expose behaviour change models applied to sustainability. Then, other complementary user models and characterisations are described, including other studies that may provide indirect, but valuable, background. Finally, the conclusions of the analysis are summarised, in order to better frame the contribution that this work provides to the research community.

### 2.1. Theoretical Frameworks of Sustainable Behaviour Change

In order to frame the relevant ideas that contextualise the present work, three relevant behavioural models that offer different approaches to frame the actions of individuals are highlighted.

Transtheoretical Model (TTM): The TTM [18] is a longitudinal model that analyses the process of change, in order to understand how it happens. It divides behavioural change into six stages: pre-contemplation, contemplation, preparation, action, maintenance, and termination. This behaviour model has been applied to boost sustainable actions and lifestyles by some scholars: He et al. [17] developed one user archetype based on this model and specific strategies to boost pro-environmental behaviour in the different stages of change; Wising, Chirez, and Adams [24] proposed a work using TTM to enhance industrial energy efficiency targeting the change of the energy culture; and in a recent work, Xiao studied how to motivate consumers in the context of sustainability by applying the TTM [25].

The theory of Values-Beliefs-Norms (VBN): VBN is a theoretical framework for sustainable behaviour based on values and norm-activation processes. The main idea of VBN is that individuals who accept a movement’s basic values believe that the valued objects are threatened. Due to this, they trust that their actions can help to restore those values and experience an obligation (personal norm) for pro-movement action, which creates a predisposition to contributing and/or giving support at different levels [26]. The values are intrinsic to the individual, but they may be aligned with the environmentalism and develop increasing beliefs that end up in the development of a pro-environmental personal norm. Beliefs appear with the acceptance of the fact that human actions harm the environment. The next step is to be aware of the consequences, followed by the ascription of responsibility. Next, the norm will be created, and the individual adopts behaviours and actions according to this norm. This approach was refined later by Stern et al. [27]. Petkov et al. applied this model in the context of pro-environmental technologies and developed a study addressing specific personalised interventions for different user types, depending on the different values outlined by Stern’s theory [28].

The Theory of Planned Behaviour (TPB): The TPB [29] is a conceptual theory that addresses intentions as the immediate antecedent of behaviour. These intentions are influenced by beliefs, attitudes, and behavioural control (the perceived self-efficacy over a specific action). This framework helps to understand how people’s behaviour can be changed or tailored with a series of predictable aspects that can influence their intended conduct. This framework has been applied to the pro-environmental context by Coskun and Erburg, in order to face the diversity of individuals in relation to sustainable behaviour [6] by developing user profiles. Furthermore, Greaves, Zibarras, and Stride analysed intentions in relation to sustainability using TPB theory, in the workplace context [30].

### 2.2. Existing User Models and Characterisations in the Context of Pro-Environmentalism

In addition to behaviour change models, some studies have explored user diversity to understand how the individuals behave. In this way, Lockton et al. developed user profiles for sustainable behaviour, taking into account the differences in behavioural traits by defining the Pinball, Shortcut, and Thoughtful user types, based on the different models of the human system [31]. Coskun and Erburg proposed another categorisation based on the TPB [19] and identified four personas in the context of the sustainability [6]. Petkov et al. [28] applied the VBN to develop a user categorisation to implement personalised eco-feedback. Moreover, He et al. [17] used the TTM for the same purpose. Cor and Zwolinski identified two types of individuals using questionnaires to measure key factors [32]. Lilley, Bailey, and Charnley developed Personas [14] focusing on the self-repair behaviour, among other factors [33]. Halko and Kientz addressed the differences in individual personalities concerning behaviour change techniques [34]; while Kaptein, Lacroix, and Saini developed profiles based on the persuasion level [35]. In a later work, they continued investigating within the field of persuasive technologies, exposing that these can be more effective if they are personalised [36]. Table 1 summarises the main idea of each proposal.

### 2.3. Other Approaches to Sustainable Behaviour

Design for Sustainable Behaviour (DfSB) [37] seeks to minimise environmental impact by framing people’s behaviour. Although not specifically focused on user diversity, Michie proposed strategies and techniques for addressing pro-environmentalism, further defining a taxonomy of techniques [38]. In addition, the same author explored the relation of behaviour change techniques with behavioural determinants [39], proposing the Behaviour Change Wheel (BCW) [40], a tool that groups 19 frameworks of behaviour change. The Theoretical Domain Framework (TDF) [41] sets an in-depth analysis of individual behaviours. This model has been applied to foster recycling behaviours [42]. Following the idea of understanding individuals better, Morgan et al. analysed the role of co-design in a large organisation [43]. They exposed ideas extracted from user studies and developed an early prototype to enhance energy efficiency in the workplace. Other related work was proposed by Yun, Aziz, and Lasternas [44], who explored the online feedback and control strategies for sustainability in the work environment. Finally, the work proposed by Bao et al. [45,46] explored the impact of quantitative and emotional feedback, highlighting the relevance of providing both feedback types. Table 2 shows the key points of the exposed works.

### 2.4. Insights Extracted from the Literature Analysis

After the review of the related work, some conclusions can be highlighted, in order to guide research into how to better understand the human factor to influence sustainable lifestyles and actions. Following the statement of Coskun and Erburg [19], there is no standard method to explore user heterogeneity, and it is complicated to extract valid knowledge. Each author applied a different approach with specific criteria in a specific context. Thus, it can be difficult to extrapolate knowledge and to further analyse the impacts of these proposals. Moreover, most of the proposals are based on a single dimension of the user. Therefore, the application of existing taxonomies may lead to several shortcomings, derived from the narrow view due to the lack of understanding of the different factors that influence the individual [7]. Finally, the difficulty of finding a dynamic categorisation of the users in the literature presents a major gap that should be tackled, in order to improve the understanding of individuals in every context.

## 3. The Proposed FOX Model

The FOX model [2] offers a framework in which the most relevant approaches to change behaviour are included, maintaining the original relations and meanings of different constructs and dimensions, while offering a general perspective on the key elements to cope with user diversity in the context of pro-environmental behaviour. This early model has been developed to be dynamic; that is, the end-user categorisation can be updated throughout time when changes in the individuals are detected. Furthermore, the model is also flexible. Thus, the dimensions of behaviour can be complemented with others (e.g., demographics) and can be prioritised, structured, and organised depending on the requirements of the application context. Nevertheless, the relations of the different dimensions of current models of behaviour change and the overall structure of these models should be taken into account, in order to avoid shortcomings. To improve the understanding of terminology, it should be clarified that the different elements involved in the behavioural theories and frameworks are called Constructs. On the other hand, the elements that compound the user meta-model are called Dimensions, which are based on the behavioural constructs in most cases. These dimensions are understood in the context of behavioural technologies addressed to enhancing awareness towards sustainable behaviour. Taking into account the analysis of the state-of-the-art, the frameworks were selected in relation to their complementary nature, selecting those that could be complemented with others, aiming at providing a more detailed conceptualisation of individuals. Another relevant factor to select the dimensions for the meta-model is related to its application to the context. All selected theoretical models have been previously applied for framing sustainable behaviour. This ensures the suitability and relevance of the selected dimensions, in relation to pro-environmentalism.

In the following subsections, the mapping of the model is explained, and the different dimensions are introduced. More detailed explanations can be found in the previous research work [2].

### 3.1. Mapping the Dimensions: The Meta-Model

**Figure 2:**
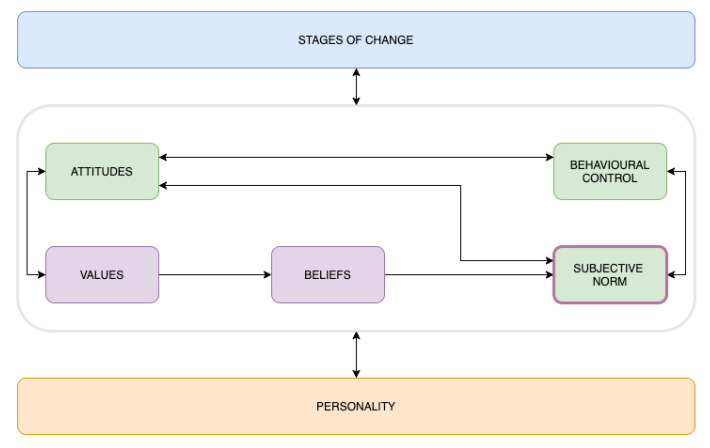
The FOX model, with the involved dimensions and relations.

The dimensions are mapped into a novel meta-model that combines the behavioural approaches found in the literature. Mixing and linking the most common theoretical models and user classifications may help to avoid a narrow perspective of each individual model by offering a more complex way to understand the behaviour. As exposed previously, different behavioural models complement each other, addressing the complexity of individuals. To better connect different items, Figure 2 illustrates the links through arrows. Following this idea, each dimension complements the others and reinforces the different approaches proposed by the theoretical frameworks.

### 3.2. Stages of Change

The TTM approaches the behavioural process by taking into account the stages of change over time [18]. Thus, Stages of Change are included in the model as a whole dimension of the user, where the phases are understood as the elements of this dimension. Following the pro-environmental approach proposed by He et al. [17], the stages of change are classified as: (1) pre-contemplation: the user has no intention to take action in the next six months; (2) contemplation: the individual intends to take action in the next six months; (3) preparation: the individual intends to take action within the next month and has taken behavioural steps to face it; (4) action: significant changes in behaviour within the past six months; and (5) maintenance and relapse: besides significant changes in the behaviour for more than six months, the person tries to sustain the acquired actions and to prevent relapse.

### 3.3. Personality

When developing behavioural technologies, the diversity among individuals with similar contexts, values, and habits (e.g., siblings or friends) implies a differential factor. Thus, diverse personalities can be determined by the acceptance and adoption of inputs. In this context, the characterisation proposed by Lockton et al. [31,47] fits well, due to the understanding of behaviour by approaching personality. This author identified three user types: (1) Shortcut users are human models that regulate their behaviour and are understood as narrowly rational users, making choices to minimise energy or cognitive expenditure; (2) Pinball users are linear human systems, implying a user who only reacts simply to inputs and does not think about any decisions; and (3) Thoughtful individuals are learning human systems—people who analytically think about what they are doing and why, being able to set and modify their own goals.

### 3.4. Attitudes

Following the ideas of the TPB, behavioural beliefs produce a favourable or unfavourable attitude toward behaviour, which changes dynamically depending on the situation and the behavioural values [48]. Attitudes are categorised incrementally, as follows: (1) No Environmental Attitude—the individual does not see benefits or immediate positive impact on sustainable behaviour; (2) Low Environmental Attitude—the individual thinks that a pro-environmental attitude may be positive, but does not see its benefits or its immediate positive impact. Besides, he/she may perceive several barriers; (3) Medium Environmental Attitude—the individual thinks that a pro-environmental attitude is positive, but perceives several barriers that obscure fully positive beliefs towards his/her behaviour change; and (4) High Environmental Attitude—the individual’s beliefs towards sustainable behaviour are at the maximum level, believing that a pro-environmental attitude is very positive.

### 3.5. Behavioural Control

Behavioural control is another construct proposed by the TPB, which is linked with the control beliefs of the user towards the specific target behaviour [48]. The classification proposed for this dimension is: (1) No Behavioural Control—the user perceives that they have no control over a specific behaviour; (2) Low Behavioural Control—the user thinks that the desired behaviour is difficult and that there are many barriers to carrying out the desired action or behaviour; (3) Medium Behavioural Control—the user thinks that it is not difficult, nor easy to achieve the target behaviour and that some barriers make the change difficult; and (4) High Behavioural Control—the user perceives that the target behaviour is easy and affordable.

### 3.6. Subjective Norm

The subjective norm is based on how sustainable behaviour is perceived by the individual and his/her context. It is related to the internal rules of the individual and his/her context. In this way, the Subjective Norm dimension is categorised as: (1) No Subjective Norm—the individual does not have subjective rules about pro-environmental behaviour and has neither internal, nor external pressure to adopt it; (2) Low Subjective Norm—the individual feels that pro-environmental behaviour is not a bad idea, but it does not feel important in his/her own life, due to the absence of a personal or social norm; (3) Medium Subjective Norm—the individual feels that pro-environmental behaviour is a positive idea, but is not fully convinced and feels relaxed about the need to adopt the behaviour; and (4) High Subjective Norm—the individual feels that pro-environmental behaviour is an obligation, due to social and personal rules.

### 3.7. Values

Values are a dimension extracted from the VBN theory, which involves a user’s personal concerns related to sustainability. Stern’s paradigm proposes four types of values [27]. Nevertheless, according to Petkov et al., values alone are not enough to boost behaviour change [28]; therefore, this dimension should be complemented with others. The classification for this dimension is: (1) Altruistic—these values are focused on avoiding threats for other people; thus, altruistic individuals are those who care about others and equality; (2) Egoistic—these values are related to the needs and wants of the individual (e.g., their gains and losses); (3) Traditional—values related to security and to maintaining the current status to preserve habits and traditions; and (4) Openness to change—values related to curiosity, involving individuals who are interested in different problems such as sustainability.

### 3.8. Beliefs

Beliefs are the last dimension extracted from the VBN, which involve the concerns related to the effects of human activity on the environment [27]. These are derived from values, following the idea that things relevant to those values are under threat. The categories are: (1) Conscious—this category involves the beliefs of the individuals who accept a new ecological paradigm; (2) Aware—this category classifies individuals who are aware of the adverse consequences for valued objects due to environmental problems; and (3) Responsible—this category involves individuals that perceive their ability to reduce the threat and, therefore, are more willing to take action and adopt sustainable behaviours.

## 4. Exploring Potential Applications of FOX

The FOX model is promising and based on other scientific contributions, but still needs to be validated, in order to demonstrate its contribution towards human–environment interaction solutions promoting sustainability. As a theoretical framework, its potential applications are diverse and can vary over time. Thus, in order to better understand the potential possibilities that this model can offer, an early study involving experts in the field of smart environments was carried out.

### 4.1. Procedure

For the development of this study, several steps were performed. First, a group of eight researchers and practitioners in the field of computer science and smart environments was selected. Recruitment was carried out by asking colleagues for voluntary participation. As the approach of this study is qualitative and exploratory, different degrees of expertise and experience were not differentiated. Due the nature of the survey, the researchers considered all kind of ideas to be welcome. Therefore, the next step was to define the method to extract the required information. As most of the participants were already familiar with the model, implementing an online survey as a method for qualitative data gathering was decided. This survey was conducted through an online Google form. For this, after introducing the FOX model, some open questions were asked about the potential applications of the framework. Taking into account the exploratory nature of the study and in order to encourage reflective answers from participants, the questions were short, general, and open, inviting individuals to explain their ideas in detail.

### 4.2. Results and Insights

**Figure 3:**
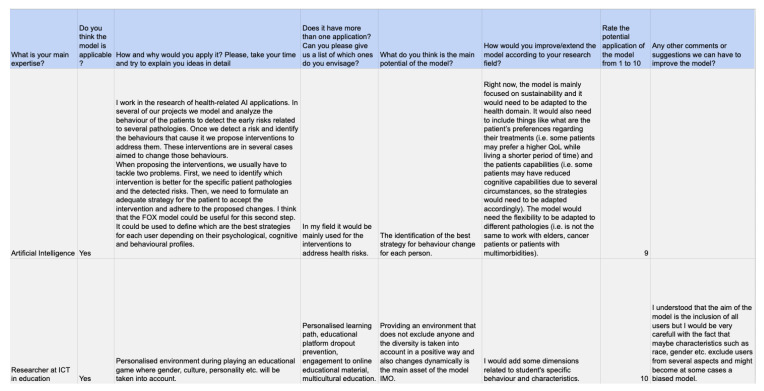
A sample of raw data obtained from the qualitative study.

After the implementation of the questionnaire, the responses were analysed and reviewed. From the gathered information, some promising ideas and relevant issues that may improve the development of the model could be extracted. In the following, the insights of each participant are described. Then, the findings are analysed, exposing the main conclusions of the study. Figure 3 shows a sample of the obtained results. The gathered data are publicly accessible. (all of the raw data are hosted and available at the following URL: shorturl.at/hANZ8) All of the respondents to the survey confirmed that the model was applicable in their field of work (e.g., Internet of Things, demand response, blockchain, or ambient assisted living). Thus, the obtained information was rich, varied, and diverse. In the following, the most relevant quotes from each participant are provided.

#### 4.2.1. Interviewee 1: Male, Post-Doctoral Researcher in Health-Related Artificial Intelligence Applications

For Interviewee 1, the main application of the model would be to implement personalised interventions to address health risks: *“When proposing the interventions, we usually have to tackle two problems. First, we need to identify which intervention is better for the specific patient pathologies and the detected risks. Then, we need to formulate an adequate strategy for the patient to accept the intervention and adhere to the proposed changes. I think that the FOX model could be useful for this second step. It could be used to define which are the best strategies for each user depending on their psychological, cognitive and behavioural profiles”*. Thus, the main potential of the model would be identification of the best strategy for behavioural change for each person. In relation to improvements, the main issues were related to the adaptation of the model to the health domain: *“It would also need to include things like what are the patient’s preferences regarding their treatments (i.e., some patients may prefer a higher quality of life while living a shorter period of time) and the patients’ capabilities (i.e., some patients may have reduced cognitive capabilities due to several circumstances, so the strategies would need to be adapted accordingly). The model would need the flexibility to be adapted to different pathologies (i.e., is not the same to work with elders, cancer patients or patients with multimorbidities)”.*

#### 4.2.2. Interviewee 2: Female, Pre-Doctoral Researcher in the Field of ICT in Education

For this person, the model could be applied to implement a *“personalised environment during playing an educational game where gender, culture, personality etc. will be taken into account*”. Other relevant application fields were also related to the educational domain: personalised learning path, educational platform dropout prevention, engagement with online educational material, and multicultural education. Regarding the main potential, in the respondent’s words: *“providing an environment that does not exclude anyone and the diversity is taken into account in a positive way and also changes dynamically is the main asset of the model”*. Regarding the improvements, she *“would add some dimensions related to student’s specific behaviour and characteristics”*. Furthermore, this participant added a relevant suggestion that should be taken into consideration: *“I understood that the aim of the model is the inclusion of all users but I would be very careful with the fact that maybe characteristics such as race, gender etc. exclude users from several aspects and might become at some cases a biased model”*.

#### 4.2.3. Interviewee 3: Male, Post-Graduate Student in Computer Science and Research Assistant in an H2020 Project Related to Energy Efficiency

The main application of the FOX model for this respondent was related to *“evaluating behaviour change interventions in work environments, such as offering food menus that include less animal meat or none at all*”. Any other applications would include similar measures or strategies that expose the individual to improve their pro-environmental behaviour. Thus, the main potential of the model was *“knowing how to help each person adopt pro-environmental behaviours without excessive profiling”*.

#### 4.2.4. Interviewee 4: Male, Researcher in the Field of Energy Efficiency

For this respondent, the application was related to energy efficiency: *“We will use the variables of the FOX model as external inputs in a causal model that will try to explain the motivations that will push a person to participate in different actions related to the use of energy. We will do so because it provides a comprehensive description of the psychological aspects that could explain the behaviour observed”*. Besides, he believed that this model can be applied in different contexts: *“we are going to assess 5 aspects: usage of energy, participation on energy efficiency or demand response actions, installation of distributed generation and the electrification of services”*. For this participant, the main potential of the proposed framework was the understanding that it offers regarding the psychological aspects of any given behaviour. Nevertheless, this participant set a relevant challenge, as *“it is difficult to define interventions (artificially set a variable to a value) according to the model”*, providing a research question to address in future work: *“Have you studied how to foster the change from one category to other in each of the variables?”*.

#### 4.2.5. Interviewee 5: Male, Researcher in the Field of the Internet of Things and Human-Computer Interaction

The fifth participant also thought that that FOX model was applicable in his field of work. In his own words: *“In my research I have to model users to understand what are their motivations to follow external guidelines or signals coming from “demand response systems” or “smart grids” to change their typical load of energy consumption. Therefore, this categorisation seems to be relevant to understand the family unit as a whole instead of individuals. I might incorporate some ideas from FOX to better understand the intrinsic and extrinsic motivations to behave in an efficient way when managing energy or to be in line with energy/load signalling”*. Regarding other possible applications of the model, the interviewee suggested the following: *“Yes, besides the energy sector. I think that the model would be beneficial in any vertical application in which the user is involved. For example, sustainable mobility, recycling, e-government or participation in citizen services”*. For this person, the main potential of the model is its versatility. Nevertheless, a relevant issue emerged from his following insight: *“Let me say also the main drawback I see. I struggle to imagine how to capture/measure in real time all the factors to infer the status of each of the constructs involved in FOX”*. This idea is in line with the answer related to improvements. The participant stated that he would improve or extend the model by *“defining factors and explicit measurements to derive the levels under each construct. I guess that questionnaires may work at the initial stage. But what if things changes dramatically? How can a system detect that? What are the implications about privacy? How the user grant access to their information to draw over its behaviour to detect the stage in which he/she is?”*.

#### 4.2.6. Interviewee 6: Male, Researcher in the Field of Artificial Intelligence Applied to Natural Language Processing and Activity Recognition

For this participant, *“In the area of active ageing, one of the objectives is to promote active ageing through a series of interventions in areas where the patient is falling behind or is beginning to show signs of problems (lack of socialisation, exercise, cognitive decline). Therefore, if these interventions could be further refined in order to provoke a more positive reception by the user in accordance with his or her profile, this would be of great help”*. Besides, for this individual, the model also could be applied *“when designing any intervention in any user/patient. That is, we can set some generic interventions but FOX could help to further refine those interventions so that they have more effect on users through ad-hoc recommendations. No matter the scope, I am focusing on active ageing but surely it can be applied to other types of users such as patients with a particular disease, children for education, etc.”*. For him, the main potential of the model was *“the facilitation of modelling different types of users taking into account aspects that are key to achieving a change in someone’s behaviour”*. Taking into account these ideas, the insights gathered in relation to improvements or difficulties were according to measurements and rules to classify users: *“What I find difficult when applying it to my field is how to classify each of the users in those dimensions. It would be interesting to be able to collect this information in the least intrusive way possible (questionnaires that are not tedious, hidden questions)”*.

#### 4.2.7. Interviewee 7: Female, Researcher and Software Developer in Energy Efficiency-Related Research Projects

The following participant also agreed on the applicability of the model. *“Lately, I’ve been working on projects which main objective is to apply a set of ICT tools to improve behavioural change. Most of the time, the software solutions that are developed, even though they are intended to a specific target group, they may not follow or may not be capable to be adapted to the specific case of each user. Therefore, the software is “static” and does not accompany the user in the change”*. Thus, she believes that the FOX model could help to improve this issue. Regarding other applications, the respondent asserted that these could be *“Tracking citizen behaviour through Social Networks (could we classify social interactions according to this model?) or tailorisation of adequate Social Actions/ICT tools needed to, for example, apply a Pay As You Throw scheme in a municipality or prevent Food Waste”*. Besides, for her, *“the main potential is that it takes into account several theoretical models to create a meta-model that analyses behavioural change from several aspects. It identifies the specific characteristics of the user along the process of behavioural change allowing to determine specific interventions customised to their needs”*. Regarding the improvements and future steps in the development of the model, her answer was in line with the other participants: *“I would improve the model maybe trying to define a set of variables that can determine how external variables for the environment can affect the intervention”*.

#### 4.2.8. Interviewee 8: Male, Researcher in the Field of Next-Generation Internet

For this participant, *“we are far away still of being able to produce fully personalised solutions, we must fit into 3-5 categories and that is it. Thus, it is necessary to find more sophisticated user models that enable to tune solutions to the attitudes, values, beliefs. Still, end users need not to be cluttered with too many extra questions”*. The framework proposed by the FOX model presents a starting point to improve the modelling process, according to these issues. For this respondent, the application of the model was linked with behaviour change interventions in the broad sense, as *“behaviour change is paramount to give place to more human-centric solutions which are more acceptable, usable and accessible for end users”*. Taking this into account, the potential of the model was linked to the personalisation of software systems and smart objects. Regarding improvements, this participant *“would analyse the impact that considering each dimension may have in the data gathering task: technical feasibility, obtrusiveness for users, ethical aspects”*.

### 4.3. Conclusions and Insights for the Potential Future Performance of the Fox Model

After the analysis of the results and understanding the gathered data, we extracted the first conclusions of the study. For this, the implications of the FOX model were exposed and defined, analysing the relevant information that should be taken into account when developing and implementing the framework.

The importance of personalised interventions. All of the respondents agreed on the applicability of the model in their own fields. Furthermore, they also agreed on the potential of the FOX model to implement personalised interventions based on specific and related dimensions. Thus, in this preliminary study, the model appears to be interesting and valuable for the research community in different contexts. Besides, the coverage of the different theoretical frameworks was valuable for Interviewee 7, reinforcing the idea of the importance of taking into account the different perspectives of behavioural frameworks to address the diversity of individuals.

Specific and complementary dimensions to foster the inclusion of individuals. In line with the previous idea, another relevant conclusion extracted was the importance of complementing behaviour-related dimensions exposed in the FOX model with non-behavioural dimensions. These need to be specific to each context and may include social and demographic data to better frame and conceptualise user profiles. Following the ideas raised by Interviewee 2, it can improve the suitability of heterogeneous user modelling, in order to avoid the exclusion of vulnerable social groups. Therefore, context is paramount when selecting dimensions, and it seems that it needs to be done without neglecting profiles (more static characterisation) and preference analysis (more dynamic and linked with context).

Flexible and updated model to accompany the individual. As Interviewee 7 exposed, one of the shortcomings of traditional user modelling is static characterisation. For that, the flexibility of the model is a relevant key point that should be addressed in any of its applications; that is, the system should be able to detect any change in its categorisation, update the status of the user profile, and offer inputs related to it. in this way, the system can accompany the individual over time and throughout the entire process of behaviour change.

Diverse context of application. Another insight extracted from the qualitative study was the applicability of the FOX model in different contexts. Although this framework was created to foster pro-environmental behaviour, Respondents 1, 2, and 6 explicitly exposed its possible application to their own fields, such as education and health. Other possible contexts of application could also be envisaged. Nevertheless, in order to apply the exposed framework to any given context, it seems crucial to study the relevant dimensions and/or variables for each context, adapting the model according to them.

Rules and measures to categorise individuals. As this preliminary model proposes a novel classification mechanism, some participants questioned the idea of how to distribute people. Thus, specific rules and measures are needed to organise individuals, according to the dimensions, and to define specific interventions for each of them. How these measures can be set implies the understanding of the requirements of the contexts and systems and, further, the study of each variable and how it was defined in the original behavioural theory. Nevertheless, this emerges as a relevant factor to develop and successfully apply a personalised user modelling framework. Seven lines of study arise from this work—one per variable—in order to analyse how to monitor and measure them, either in the context of smart environments or using self-evaluation tools.

Neither intrusion nor tedious systems for data gathering. In following with the previous idea, the data gathered for the modelling should be done with non-intrusive systems that provide a good experience. Thus, the relevance of the design-specific and non-intrusive data gathering tools emerges as a need to further develop the FOX model. Nevertheless, how the data can be gathered depends on the technological capability and the context. Although gathering the data through questionnaires and other traditional tools can be very tedious, in most cases, data collection can now be done through smart devices and specific sensors located to capture information with no intrusion, while taking into account the privacy of the individual.

Avoid biases to foster diversity. As a final conclusion, a relevant insight from Interviewee 2 should be taken into account: As the FOX models offer early user classification to understand the heterogeneity of individuals better, it is highly important to be careful with the fact that the inclusion of some characteristics (e.g., demographics such as gender, race, country, and so on) may bias the model. Thus, the inclusion of complementary dimensions and the definition of the specific rules for each dimension should be studied and analysed in detail, in order to ensure that the model does not include any bias.

## 5. Applying the FOX Model

**Figure 4:**
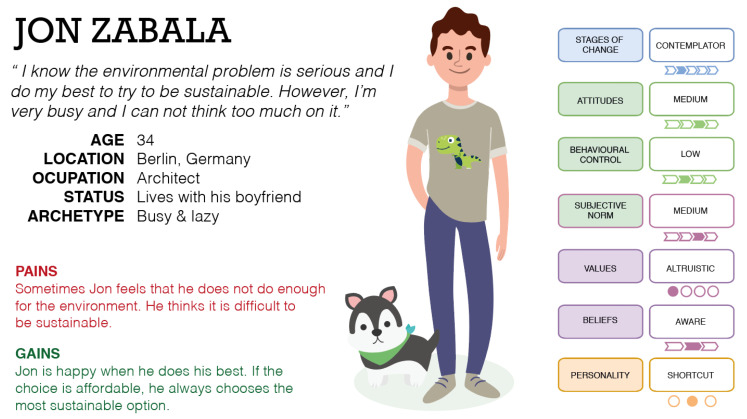
Jon and the main characteristics that are included in the profiling at the initial stage.

**Figure 5:**
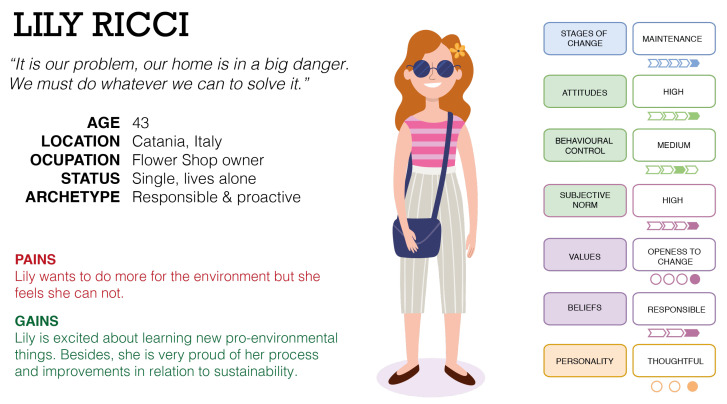
Lily’s Persona profile and her main characteristics at the initial stage, according to the FOX model.

**Figure 6:**
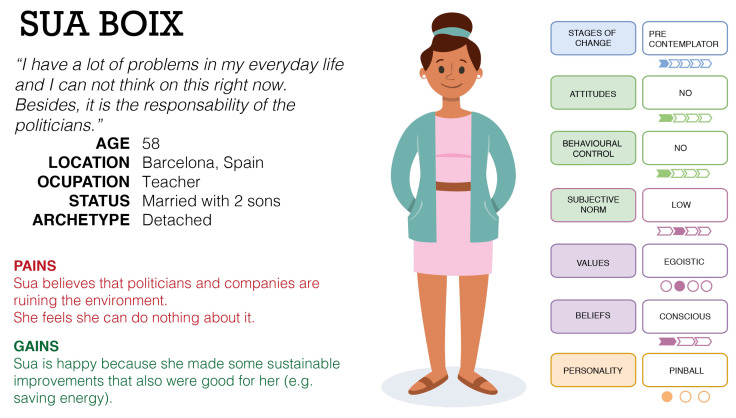
Sua’s Persona profile and her main characteristics at the initial stage, according to the FOX model.

Once the main potential applications were explored, we sought to understand how the model can be applied to tackle user heterogeneity when influencing sustainable actions, when specific user profiles are defined. Thus, a simple textual example of three possible user archetypes using a Persona [14,15] is used in the following; these profiles are described below. In addition, Figure 4 shows the Persona of Jon, Figure 5 the Persona of Lily, and Figure 6 the Persona of Sua.

### 5.1. Developing User Profiles: Jon, Lily, and Sua

In the following, three Personas are exposed by defining and understanding all the relevant aspects to envision the user archetype as a real individual. These profiles were developed taking into account the different personalities proposed in the FOX model. Thus, Jon is a Shortcut user, who makes choices to minimise effort; Lily is a Thoughtful user, who analytically thinks about what she is doing and why; and Sua is a Pinball user, who reacts simply to inputs, doing the same thing each time the same stimulus is applied. These user profiles have been categorised in the context of the FOX model, complementing the dimensions with demographic data and other insights to enrich the modelling and to take into account other relevant dimensions that complement and contextualise the archetypes.

#### 5.1.1. Jon

Jon lives in Berlin with Peter and Zero (their dog). He is a 34-year-old architect who recently started a new job in a very prestigious architecture studio. He is a hard-working person who is always worried about being efficient and productive in his life. He is very familiar with technological devices and gadgets. He has been a fan of the Apple brand for a long time. At present, he owns two Macs, an iPhone, an Apple Watch, AirPods, and an iPad. He likes rock music and going to music concerts. Jon is not a sporty person, but he tries to stay active in his everyday life. Therefore, he goes walking whenever possible and goes hiking on the weekends. Since he was a child, he has loved dinosaurs. He has a great collection of books and other related stuff. His perfect weekend is a concert with friends on Saturday and watching Netflix and relaxing on Sunday. Jon is conscious of the environmental situation and its importance. He cares about the situation of people in danger, and most of his friends are very active in this field. Indeed, pro-environmentalism is a common topic in his everyday life. Due to this, Jon somehow aspires to improve his lifestyle according to his concerns. For example, he eats less meat, and he tries to reduce his waste output. Nevertheless, as he has many things on his mind, he does not make any relevant effort to improve the impact of his actions. He often thinks about it and feels guilty, but due to the fact that he finds it complicated to make any meaningful impact on the environment, he does not behave fully responsible in terms of sustainability.

#### 5.1.2. Lily

Lily lives in Catania, Italy. She is a 43-year-old woman who lives in a small flat in the city centre together with her two cats, Milos and Alfred. She loves her small flower shop and works hard on it. She is an open-minded person who is calm, optimistic, and polite. She enjoys everyday contact with people and loves working with flowers and plants and being in natural environments. She uses social media in her daily life, mostly to promote her business and to deal with customers. Indeed, she prefers personal relationships. She practices yoga and Tai-Chi once a week. She reads a lot; most of the books are historic and noir novels, and as she is a dreamer, she enjoys visualising herself in these adventures. Lily is a convinced vegan, although she does not like persuading others about it. Her perfect weekend is travelling outdoors to discover landscapes by walking in the middle of nature, followed by having a coffee and cake in a nice place. Lily is very aware of the environmental situation. She feels responsible for it, and she is very active in doing her best to reduce her environmental impact. For Lily and her friends, being environmentally conscious is very important; therefore, they always make the most environmentally friendly choices when eating, travelling, buying groceries, and so on. She always reflects on her actions and tries to improve her behaviour. Nevertheless, she feels that some environmental issues are out of her control, and she feels frustrated about it.

#### 5.1.3. Sua

Sua (58) lives in Barcelona with her husband Josu. They have two sons, Mikel (29) and Aitor (33), who moved out a few years ago, but live near them, at their own places in Barcelona. Sua is a teacher at a high school with 15–16-year-old students. She has worked there for more than 20 years, and she is happy there, although she thinks very often about retirement. Although Sua and her family do not have big problems, Sua is always worried and concerned about her husband and her sons. Sua and Josu love going to the beach. They own a house in a small village near the sea, and they go there very often. Sua likes cooking and gardening, growing vegetables in the small garden they have in their country house. Her perfect weekend is going to the beach early in the morning, then preparing and enjoying a big lunch for all of her family with her own raised vegetables. Sua is not really concerned about the environmental situation. She recognises that it is a problem, but she does not feel it is her problem. She believes that this situation is a direct consequence of the poor management of politicians, and she does not feel responsible for it. As she is constantly worried about her family’s economics, she is worried about the specific behaviours that have a direct impact on them, such as the monthly bills. Nevertheless, as she does not think about her actions, she has made some improvements (due to her sons) without noticing them.

### 5.2. Understanding Actors in Their Context

**Figure 7:**
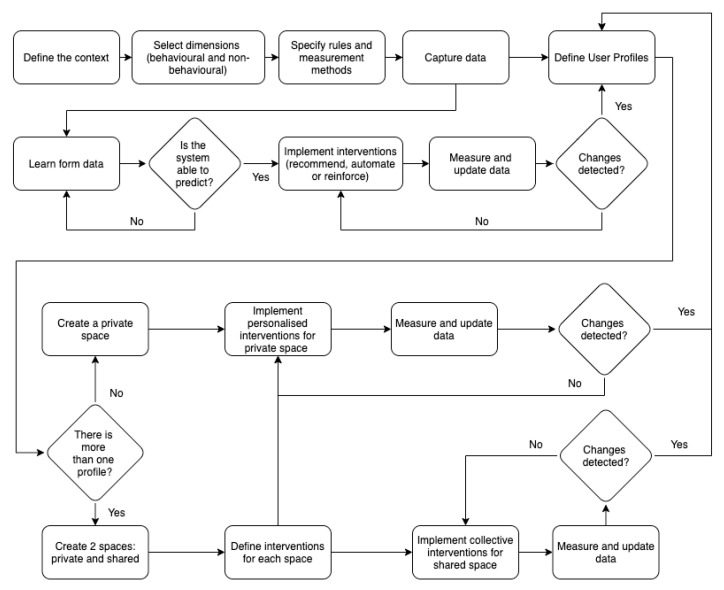
The workflow defined to identify the main stages in the application of the FOX model.

**Figure 8:**
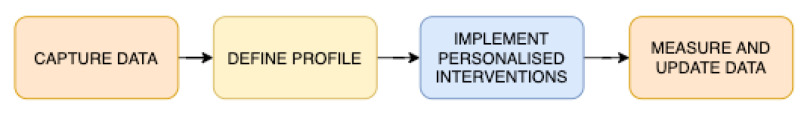
The key stages of Jon’s scenario.

**Figure 9:**
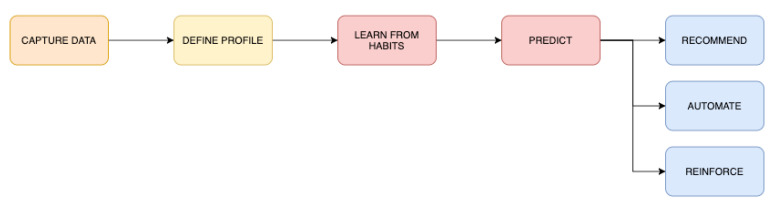
The key stages of Lily’s scenario.

**Figure 10:**
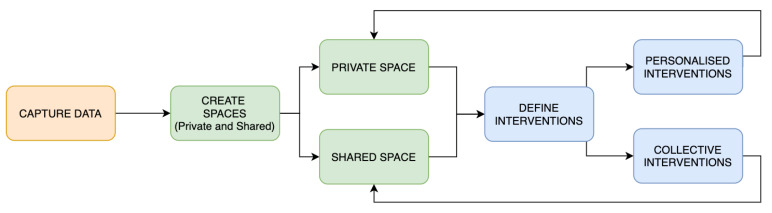
The key stages of Sua’s scenario.

Once user profiles are defined, the next step is to underpin how these archetypes perform their sustainable behaviour by applying the FOX model in the context of smart environments. For that, the first step is to set the main stages and actions of the implementation process to identify the critical areas. Figure 7 shows the workflow defined to apply the FOX model. This tool will help to detail the specific tasks and stages of the process to gain knowledge on how the system will work. Based on the workflow, three specific scenarios are developed, one for each user model: (1) The scenario of Jon is contextualised in his workplace. In this open space, the main devices are shared with other colleagues, but they have a smart system that can detect individual actions and give personalised inputs according to them. Figure 8 exposes the performance of the model in this case. The main device involved in this context is an app that offers information and other features. As Jon’s workplace is a smart office, this app is connected to the different sensors of the office. (2) The context of Lily involves her home. She has just one smart device, a smart meter, which can learn from Lily’s behaviour and predict her actions, reinforce her behaviour, offer recommendations, and suggest automation strategies. Figure 9 shows the workflow of this scenario. (3) The last scenario involves Sua and her family. Their context involves a smart home (an individual space with a smart system installed), where the key factor is the inclusion of different profiles, offering common interventions to reinforce the group, as well as personalised inputs, according to the different user profiles. In this way, all the individuals have a common goal while preserving their personal preferences. Thus, they will feel a part of the group while maintaining their individuality. The devices involved in this scenario are mainly the app and other home appliances connected to the Internet, such as the vacuum cleaner, the lights, and the thermostat. Figure 10 visualises the process of this context. In the following lines, the three scenarios are described, aiming at understanding how the individuals would perform their activities, taking into account the framework proposed by the FOX model.

#### 5.2.1. Jon and His Workplace

At Jon’s new job, they are very concerned about the environmental impact of their buildings. They try to reduce the energy demands of the buildings they design and try to consider the sustainability of their workplace. Due to this, they have installed sensors across the building to capture data and optimise the usage of processes and devices. Furthermore, they created a smartphone app named Foxi, which offers feedback and data about environmental concerns to motivate and influence workers. This application includes four main sections: (1) Information, where different data and information related to sustainability are provided; (2) Control, where the user can connect to devices and control them; (3) Social, where an individual can interact with other people; and (4) User settings, in order to manage user profiles and to personalise some features. This application was inspired by a prototype presented in a previous research work [8], where user diversity was addressed by offering personalised strategies and techniques. Nevertheless, with the inclusion of the dimensions presented in the FOX model, the Foxi app could offer more complex and personalised strategies than the initial prototype.

Understanding Jon. In his second day at work, at lunchtime, Jon decides to download and use the app that his company developed, in order to boost his awareness about the importance of sustainable behaviour. To create his profile, he answers some questions in the app. Next, he goes to his desktop and works normally. In the following days, the smart environment of the workplace obtains relevant data about Jon, and by comparing it with the data of the app, a preliminary categorisation is implemented: (1) Jon is categorised as a contemplator, as he recognises some problematic behaviours and thinks of how to improve them; (2) his values are categorised as altruistic; (3) Jon has been included in the aware category, taking into account his environmental beliefs; (4) the Subjective Norm of Jon is medium: in his work environment, the norm is very high, but in other environments (e.g., at his home), there is not a pro-environmental norm; (5) the attitudes of Jon regarding sustainable behaviour are categorised as medium; (6) the behavioural control of Jon in the context of his workplace is low; and (7) the personality of Jon is categorised as Shortcut.

Implementing interventions. Once Jon has been located in the dimensions of the FOX model, specific interventions can be implemented to improve his pro-environmental lifestyle awareness. Taking into account the existing categorisation of Jon, the following strategies are implemented: (1) In order to motivate Jon to change to the next stage, preparation, the application gives information about the benefits of being sustainable. (2) The Foxi app offers a data visualisation of the impact of climate change on other people in different parts of the world, in order to enhance the importance of sustainability values. Jon can also access other relevant data related to other factors (e.g., economic information and information about the impact on animals and plants). (3) Taking into account the data gathered by specific sensors in Jon’s workplace, they decide to implement an awareness campaign showing the data of the average energy waste of a single individual at work and tips to avoid it, aimed at motivating workers to be responsible with their own actions. (4) To boost the Subjective Norm in relation to sustainability, the Foxi app shows the profile and data of prominent pro-environmental people (in this case, famous architects). Further, it offers a social network to boost relationships with other people with similar interests. (5) Aiming at enhancing pro-environmental attitudes, Jon’s company showcases a documentary film about the impact of climate change. (6) The Foxi app offers reminders that can be personalised by Jon. In this way, he does not forget to turn off his devices when leaving the office. Finally, (7) as a Shortcut user, he makes the effortless choice. Therefore, the Foxi app has every single process set by default (favouring the most pro-environmental options).

Updating the model. Once the interventions have been implemented, the next step is to compare whether there have been improvements in motivation about sustainable behaviour. Through a short survey (using the Foxi app) and the data gathered by the smart environment, the new status is recognised, and the model is updated. (1) After the interventions, Jon started to plan new pro-environmental actions. Therefore, the model updated this dimension, and now, Jon is in the preparation stage. (2) The values of Jon were aligned with pro-environmental issues, and now, the altruism of Jon involves sustainability concerns. Besides, he has consulted data related to expenses and money saving. Therefore, the Foxi app improved the visibility of these data, and the categorisation of Jon changes from the altruistic to the egoistic dimension, depending on the most-viewed data. (3) Jon has taken responsibility for the environmental problem. Therefore, his categorisation changed to responsible. (4) After the interventions, the Subjective Norm of Jon rose and is now categorised as high. (5) In the update of the model, the attitude of Jon about sustainability rose, and Jon now has a high pro-environmental attitude. (6) After the interventions, the behavioural control of Jon was categorised as medium. (7) Jon maintains the Shortcut behaviour, most of the time, but the system detected that (mostly on the weekends), he checked other data (such as his historic energy expense). Therefore, in those days, the app will give feedback to boost his knowledge and to help him to learn about his behaviour.

#### 5.2.2. Lily at Her Home

Lily is very aware of her pro-environmental behaviour. She always tries to make the most sustainable choices, and therefore, she wants to manage the energy expenditure of her house efficiently. For this reason, following the advice of a friend, she bought a smart meter. This device is connected to the main electricity board of her house and gathers information about her electricity consumption. It is also connected to the Internet and has a linked mobile application to visualise and manage some settings. In the words of Lily’s friend, “ *this will help you a lot to understand your consumption patterns and to save energy accordingly* ”. With this idea in mind, she acquired the device, and after some difficulties and two calls asking her friend for help, she finally succeeded in installing it and making everything work.

Capturing and learning. When opening the application for the first time, Lily introduced some data about herself and her lifestyle. At this moment, her profile was created (combining the dimensions proposed by the FOX model with other demographic data) and saved into the server. Then, Lily can tap into some sections, researching and seeking some information. She can visualise the energy consumption of her house and also other relevant information that she did not know. She was very happy, as she could now understand her energy consumption and waste better. Besides, she learned when the energy was less expensive and greener. In the following days, Lily continued checking the app for information. Throughout the process, she learned a great deal, both about general sustainability tips and about her own consumption patterns. She also became very active on the social network of the app. During these days, the system captured data and updated the model periodically, learning about Lily’s lifestyle, preferences, and consumption patterns.

Predicting and forecasting behaviour. After a few days, the system learned enough information and was able to make predictions. Lily is still interested and tries to learn, but as she is a very regular and pro-environmental person, her chances for making improvements are minimal. One day, she received a notification from the app: “Predictions are now enabled!”. She taps on it and discovers a new section. Here, she sees a historic visualisation of her past consumption. She also finds a forecast of her expected future consumption and a short summary of future predictions related to her behaviours and actions. This was a nice surprise for Lily, as now she can learn about the future and about the impact of her actions.

Recommending, reinforcing, and automating. Once the system is able to predict the actions of Lily, some new interventions can be offered, in order to improve her motivation and to support her pro-environmental behaviour. These are grouped into three main actions: recommend, reinforce, and automate. Based on the predictions created, taking into consideration the FOX model, the system can recommend personalised interventions to boost the motivation of the individual. These can be varied and diverse; for example, information related to Lily’s values and beliefs, ideas and strategies to improve her behavioural control, and other data related to the impact of her actions can be offered. The system can also reinforce Lily’s future behaviour by offering rewards and other motivational inputs. Finally, taking into account the predictions of the system, some actions may be automated. For example, as Lily spends most of her day at work, the system can suggest to her to automatically switch off the devices that are not needed when she is out, turning them on just before her arrival.

#### 5.2.3. Sua and Her Family

Sua is on vacation with her husband and sons. They just arrived home from the beach. While some of them go to their bedrooms to change their clothes, the others go to the kitchen to prepare some snacks and drinks while they wait their turn for the shower. They speak about what they are going to prepare for lunch while they check what is available in the fridge. At some point, Sua remembers that the tomatoes in the garden are at the perfect point to be harvested, so she goes outside to check them. While walking back to the kitchen, Sua checks her phone. She finds a notification offering ideas for preparing dishes with home-grown vegetables, so she opens the app and searches for tomato salad recipes. Now, in the kitchen again, she chats with her son about the app. He reminds Sua that the app is connected to other devices, such as the lamp or the coffee maker. These devices were a gift from last Christmas from Sua’s sons to their parents. As they know that their parents love being at that house and taking care of the garden, they invested their money to transform the beloved beach house of their parents into a smart home.

Creating profiles and spaces. When Mikel and Aitor installed the devices, they also installed the app in their parent’s smartphones. Each member of the family created a profile, and the sons taught their parents how to quickly use the management system. Josu liked the system and got used to it very fast. It was quite easy for Sua as well, but she did not really care much about it; so, she ended up forgetting what she had learned. Nevertheless, Josu was a frequent user of both the app and the connected smart devices; for example, Josu had automated the vacuum cleaner, and he checked the app very often. His preferred feature was space management: as he is always the one managing the gadgets of the house, he can check such common features as their energy and water expenditure and other shared devices, as well as checking his private area, where he can find personalised information and content.

Analysing similarities and differences. While Sua is chopping tomatoes to prepare the lunch, Josu reminds her about the fancy functions that their smart home has. Sua notices some improvements; for example, she acknowledged that the automatic vacuum cleaner is a really nice device that allows her to not worry about cleaning the floor. At that point, Josu starts speaking about other things that Sua did not notice. Thus, she checks the app in her phone. In her private area, she finds out that her stats and information are different from what Josu had shown her in his phone. Indeed, she can edit the information she can see, according to her preferences. in this way, she sets the feature to see how much money they save, regarding their pro-environmental behaviour.

Common and personalised interventions. The family is enjoying lunch after their beach day. Sua continues chatting about the re-discovered app, as she is very happy with the new functions she has learned and is really excited as she can manage money better now. While their sons are joking about the fact that it took some months to discover the benefits of their present, Sua wonders about the “Challenge” feature. They explain to her that this is a feature that involves all of the family members. It is a competition among different groups of user, where the most pro-environmental group wins. Sua checks the ranking, and the family starts speaking about their possibilities in the challenge. Sua is happy because all the family is excited about a common goal, which she likes. She invites her family to participate in the challenge together; however, she is worried, as she liked the other personalised features she discovered, such as the “recipe advisor” and the “money saving calculator”. She asked her sons: *“So... If I join a challenge with you, will I lose my private settings?”*. They answer: *“No mum, the system offer us common features as a family, but you still have your own space, based on your preferences and activities”*. With this idea in mind, Sua was happy because she could have a common activity with her family where of all the different members are included, but still keep her own space to check her personalised content.

### 5.3. Conclusions and Insights

From the development of the exposed Personas and/or user profiles, some insights that can serve to guide future research work can be extracted. First, it can be ensured that the developed user archetypes provide valuable information and may improve the understanding of the real users. Besides, the complexity of the individual is addressed through the inclusion of the dimensions proposed in the FOX model, in combination with other demographic attributes. In addition, the proposed profiles could help to identify the emotions and feelings related to pro-environmental behaviour. Finally, the contextualisation of individuals in their own context, the novel categorisation framework, and the identification of specific barriers (see Figure 4, Figure 5 and Figure 6, where the barriers are included under the “Pains” section) are other benefits. From the proposed case studies or scenarios, the main contribution may be the understanding of how each user archetype performs his/her behaviour in his/her own context. This idea could help researchers and practitioners improve their understanding of the performance of individuals in the context of sustainability. Through these case studies, the performance of the model can be exposed: in the first example (Jon), the performance of the model was centred on the implementation of personalised interventions; in the second case, the performance was focused on behaviour prediction and forecasting; finally, in the third case, the performance of the model was centred on the management of different profiles and contexts (combining private and shared spaces).

## 6. Discussion

In this paper, the potential applications of the FOX model were explored. For this purpose, a qualitative survey with experts was performed (Section 4), with the framework applied to the development of three user archetypes (Section 5.1) and case studies (Section 5.2). This follows the line of other authors, such as He et al. [17], Coskun and Erburg [19], and Lockton et al. [31]. Nevertheless, as the FOX model combines four behavioural frameworks, it may cover several complementary points of view that can help to understand, in a more accurate fashion, the complexity of the individual in different contexts. Of course, these characterisations could be improved by understanding and embracing other relevant issues, such as the specific barriers inherent to each profile. After the development of three Personas (or user profiles), in order to gain knowledge about the behaviours of individuals in their own context and to improve understanding of the performance of the model, three use cases were developed, exploring three different modes of implementation of the FOX model: (1) personalised interventions, (2) prediction and forecasting, and (3) individual and collective interventions. The difference between user archetypes or Personas and case studies is that the former explores an individual’s universe and his/her complexity, while the latter explores how these archetypes perform their behaviours and interact with the system in any given context. In this way, the development of Personas may help to better understand individuals, while the development of the case studies may help to comprehend how these user archetypes interact with the system in their everyday life. Furthermore, although more research is needed to validate the findings, the possibilities the FOX model offers can be envisaged through these scenarios. Furthermore, the implementation of the ideas can be conceptualised in an early stage of the process, in order to test whether an idea or intervention works in a given context, before investing resources into it. This is in line with the BCW [40], a behavioural approach where a variety of dimensions are covered. Nevertheless, the work presented in this paper addresses the flexibility of individuals, proposing a dynamic and multilevel characterisation that may help to improve the understanding of the complexity and variability of people.

Another critical factor that should be considered is the importance of complementing the dimensions of the model with other specific data. From the results of the questionnaire, we conclude that the dimensions should vary depending on the context of the application, complementing the core dimensions of the model with other information such as demographic variables. Besides, the proposed case studies show also how the usage of non-behavioural dimensions is a paramount issue. This idea may enhance the inclusion of diverse profiles, especially of the most vulnerable ones. However, how the complementary variables are gathered and selected must be carefully studied to avoid biases in the user modelling process.

Additional relevant topic that should be further investigated relates to the way that rules and measures for classifying individuals should be set up. As the results of the qualitative study show, in order to implement the FOX model in the user modelling process, specific measures are needed to include the individual in each category, depending on their characteristics. For this, the behavioural theories and frameworks must be taken into account, in order to maintain the original settings and classification procedures. Nevertheless, as some respondents stated, data gathering is typically tedious. Therefore, complex questionnaires should be avoided, and the required data should be captured using tools and methods that provide a positive experience. Thus, the rules of each dimension and how the data are gathered should be studied in the specific context of the application. Furthermore, available technologies must be also taken into account; for example, in smart environments, data gathering can be done through non-intrusive methods, by using sensors and other smart devices that maintain the privacy of people.

Finally, from the qualitative study, we also extracted how the FOX model can be applied to different contexts. From health to education, the model may be implemented to cover the diversity of individuals when developing interventions in any target population. Nevertheless, as previously indicated, the selection of the dimensions, the development of specific rules and measures, and the implementation of data gathering tools must be carefully considered, in order to adapt and apply the model successfully.

**Figure 11:**
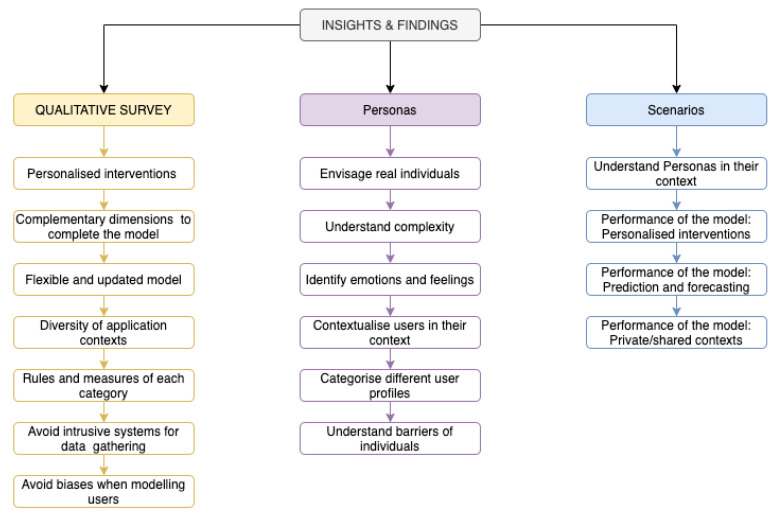
The main insights and findings of the research work presented in this paper.

To provide a summary of the main insights and findings of the presented research work, Figure 11 exposes the specific ideas and contributions obtained from each stage of the research process: the qualitative survey explained in Section 4 and the case studies developed in Section 5 (Personas and scenarios).

## 7. Conclusions

In this paper, a holistic meta-model (named FOX) that classifies individuals by taking into consideration the behavioural dimensions related to pro-environmental behaviours was presented. The FOX model seeks to consider the heterogeneity of the individuals through a dynamic categorisation method with the objective of offering a flexible framework. Aiming at understanding how the model could perform to boost an individual’s behaviour, an exploratory qualitative experiment was conducted. In this study, eight experts in the computer science and smart environments fields reflected on the fox model and its potential uses. Then, the mechanisms and processes to apply the FOX model were detailed by defining a guiding procedure (workflow to follow) in order to identify the key tasks and actions needed to put FOX in practice. Moreover, seeking further applications and new insights, this model was applied to the modelling of three specific user archetypes, in order to understand the different concerns of the individuals in relation to sustainability. Furthermore, three case studies were exposed, each of them involving one user profile in the context of smart environments. These use cases demonstrated how the model can perform in different scenarios. From the presented work, it can be concluded that the FOX model is promising, as it is flexible and extendible, for researchers and practitioners in the field of behavioural research, as well as for computer scientists aiming to better understand users. Moreover, the results of the study show that the model may be applied in the context of smart environments, in order to foster pro-environmental behaviour. Furthermore, the application of the framework to other potential contexts (e.g., health and education) is feasible, by including variables and dimensions specific to the context. The application of the FOX model when defining user profiles and scenarios could provide valuable knowledge for improving the understanding and inclusion of individuals when designing and developing technologies. Although further research is needed to consolidate the findings and insights presented in this paper, the present work sets a starting point to develop a human-centric approach and to target user needs when coping with forming or increasing pro-environmentalism and eco-awareness. Finally, from the present work, some improvements and reflections could be extracted, setting specific future research lines. This future work should be evolved, in order to extract more knowledge and consolidate the findings presented in this paper. These next steps are: (1) define the tools that allow for measuring dimensions, stages, and characteristics of the meta-model; (2) test the model with different groups and individuals (i.e., specific target users, researchers, practitioners, and so on), in order to include valuable insights from different points of view and to validate the proposed framework; (3) complement the model with other specific and context-related dimensions that may influence user behaviours; and (4) explore the application of the model with different methods in different processes, in order to better understand its possibilities. Finally, another iteration of the whole research process (see Section 1.1) may be implemented, in order to refine and validate the knowledge exposed in the present work.

## Figures and Tables

**Table 1:** Existing user models and characterisations in the context of pro-environmentalism. TTM, Transtheoretical Model; VBN, Values-Beliefs-Norms.

Authors	Description and Key Points
He et al., 2010 [17]	Application of TTM to define interventions for one specific Persona.
Halko and Kientz, 2010 [34]	Understanding differences in personality, in relation to behavioural techniques.
Kaptein, Lacroix, and Saini, 2010 [35]	User profiles linked with the persuasive level and personalised inputs.
Lockton et al., 2012 [31]	Definition of three user profiles in relation to behavioural traits.
Petkov et al., 2012 [28]	Application of VBN, identifying profiles to address personalisation.
Lilley, Bailey, and Charnley, 2013 [33]	Development of three Personas and specific strategies for them.
Cor and Zwolinksi, 2014 [32]	Identification of two types of individuals to set design interventions.
Coskun and Erburg, 2016 [19]	Identification of four personas based on the TPB.

**Table 2:** Other approaches to sustainable behaviour. DfSB, Design for Sustainable Behaviour; BCW, Behaviour Change Wheel; TDF, Theoretical Domain Framework.

Authors	Description and Key Points
Michie, 2008 [39]	Exploration of behaviour change techniques with behaviour determinants.
Lilley, 2009 [37]	Definition of DfSB, a framework to address behavioural interventions.
Michie, 2011 [38],	Definition of a taxonomy of behaviour change techniques.
Michie, 2011 [40]	Development of BCW, a tool to select the best behavioural strategy.
Gainforth, 2016 [42]	Application of TDF to foster recycling behaviours.
Bao et al., 2016 [46]	Analysis of quantitative and emotional eco-feedback.
Atkins, 2017 [41]	Definition of TDF, a framework addressing behaviours of individuals.
Yun, Aziz, and Lasternas, 2017 [44]	Analysis of online feedback and control strategies in the workplace context.
Morgan et al., 2018 [43]	Research work about co-design in a large organisation to boost sustainability.
Bao et al., 2019 [45]	Analysis of emotional eco-feedback.

## References

[1] Sánchez-Corcuera R., Nuñez-Marcos A., Sesma-Solance J., Bilbao-Jayo A., Mulero R., Zulaika U., Azkune G., Almeida A. (2019). Smart cities survey: Technologies, application domains and challenges for the cities of the future. Int. J. Distrib. Sens. Netw..

[2] Irizar-Arrieta A., Retegi A., Casado-Mansilla D., Laschke M., López-de Ipiña D. FOX: A flexible and heterogeneus mixed user model to address sustainable behaviour in smart wnvironments. Proceedings of the Multidisciplinary Digital Publishing Institute Proceedings.

[3] Mankoff J.C., Blevis E., Borning A., Borning A., Friedman B., Fussell S.R., Hasbrouck J., Woodruff A., Sengers P. Environmental sustainability and interaction. Proceedings of the ACM CHI’07 Extended Abstracts on Human Factors in Computing Systems.

[4] DiSalvo C., Sengers P., Brynjarsdóttir H. Mapping the landscape of sustainable HCI. Proceedings of the ACM SIGCHI Conference on Human Factors in Computing Systems.

[5] Bravo J., Fuentes L., de Ipina D.L. (2011). Theme Issue: “Ubiquitous Computing and Ambient Intelligence”.

[6] Coskun A., Erbug C. User diversity in design for behavior change. Proceedings of the DRS.

[7] Hekler E.B., Klasnja P., Froehlich J.E., Buman M.P. Mind the theoretical gap: Interpreting, using, and developing behavioral theory in HCI research. Proceedings of the ACM SIGCHI Conference on Human Factors in Computing Systems.

[8] Irizar-Arrieta A., Casado-Mansilla D. Coping with user diversity: UX informs the design of a digital interface that encourages sustainable behaviour. Proceedings of the 11th Multi Conference on Computer Science and Information Systems.

[9] Irizar-Arrieta A., Casado-Mansilla D., Retegi A. Accounting for user diversity in the design for sustainable behaviour in smart offices. Proceedings of the IEEE 2018 3rd International Conference on Smart and Sustainable Technologies (SpliTech).

[10] Buchanan E.A., Hvizdak E.E. (2009). Online survey tools: Ethical and methodological concerns of human research ethics committees. J. Empir. Res. Hum. Res. Ethics.

[11] Evans J.R., Mathur A. (2005). The value of online surveys. Internet Res..

[12] Schwandt T.A., Gates E.F. (2018). Case study methodology. Sage Handb. Qual. Res..

[13] Hosono S., Hasegawa M., Hara T., Shimomura Y., Arai T. (2009). A methodology of persona-centric service design. Proceedings of the 19th CIRP Design Conference–Competitive Design.

[14] Blomkvist S. (2002). Persona—An overview. Retrieved Novemb..

[15] Pruitt J., Grudin J. Personas: Practice and theory. Proceedings of the 2003 Conference on Designing for User Experiences.

[16] So C., Joo J. (2017). Does a persona improve creativity?. Des. J..

[17] He H.A., Greenberg S., Huang E.M. One size does not fit all: Applying the transtheoretical model to energy feedback technology design. Proceedings of the ACM SIGCHI Conference on Human Factors in Computing Systems.

[18] Prochaska J.O., Redding C.A., Evers K.E. (2015). The transtheoretical model and stages of change. Health Behav. Theory Res. Pract..

[19] Coskun A., Erbug C. Exploring and communicating user diversity for behavioural change. Proceedings of the 2016 Design Research Society 50th Anniversary Conference.

[20] Madsen S., Nielsen L. (2009). Exploring persona-scenarios-using storytelling to create design ideas. Proceedings of the IFIP Working Conference on Human Work Interaction Design.

[21] Madsen S., Nielsen L. Using storytelling to improve scenarios. Proceedings of the IADIS International Conference Information Systems.

[22] Coskun A., Zimmerman J., Erbug C. (2015). Promoting sustainability through behavior change: A review. Des. Stud..

[23] Irizar-Arrieta A., Gómez-Carmona O., Bilbao-Jayo A., Casado-Mansilla D., Lopez-De-Ipina D., Almeida A. (2020). Addressing behavioural technologies through the human factor: A review. IEEE Access.

[24] Wising U., Chirez S., Adams B. Improving industrial energy efficiency by changing the energy culture. Proceedings of the ECEEE Industrial Summer Study Proceedings.

[25] Xiao J.J. (2019). Developing action-taking programs in sustainable consumption education: Applying the transtheoretical model of behavior change (TTM). SSRN.

[26] Stern P.C., Dietz T., Abel T., Guagnano G.A., Kalof L. (1999). A value-belief-norm theory of support for social movements: The case of environmentalism. Hum. Ecol. Rev..

[27] Stern P.C. (2000). New environmental theories: Toward a coherent theory of environmentally significant behavior. J. Soc. Issues.

[28] Petkov P., Goswami S., Köbler F., Krcmar H. Personalised eco-feedback as a design technique for motivating energy saving behaviour at home. Proceedings of the ACM 7th Nordic Conference on Human-Computer Interaction: Making Sense Through Design.

[29] Ajzen I. (1991). The theory of planned behavior. Organ. Behav. Hum. Decis. Process..

[30] Greaves M., Zibarras L.D., Stride C. (2013). Using the theory of planned behavior to explore environmental behavioral intentions in the workplace. J. Environ. Psychol..

[31] Lockton D., Harrison D., Stanton N.A. (2012). Models of the user: Designers’ perspectives on influencing sustainable behaviour. J. Des. Res..

[32] Cor E., Zwolinski P. (2014). A procedure to define the best design intervention strategy on a product for a sustainable behavior of the user. Procedia CIRP.

[33] Lilley D., Bailey V., Charnley F. (2013). Design for Sustainable Behaviour: A Quick Fix for Slower Consumption?.

[34] Halko S., Kientz J.A. (2010). Personality and persuasive technology: An exploratory study on health-promoting mobile applications. Proceedings of the International Conference on Persuasive Technology.

[35] Kaptein M., Lacroix J., Saini P. (2010). Individual differences in persuadability in the health promotion domain. Proceedings of the International Conference on Persuasive Technology.

[36] Kaptein M., Markopoulos P., De Ruyter B., Aarts E. (2015). Personalizing persuasive technologies: Explicit and implicit personalization using persuasion profiles. Int. J. Hum. Comput. Stud..

[37] Lilley D. (2009). Design for sustainable behaviour: Strategies and perceptions. Des. Stud..

[38] Michie S., Ashford S., Sniehotta F.F., Dombrowski S.U., Bishop A., French D.P. (2011). A refined taxonomy of behaviour change techniques to help people change their physical activity and healthy eating behaviours: The CALO-RE taxonomy. Psychol. Health.

[39] Michie S., Johnston M., Francis J., Hardeman W., Eccles M. (2008). From theory to intervention: Mapping theoretically derived behavioural determinants to behaviour change techniques. Appl. Psychol..

[40] Michie S., Van Stralen M.M., West R. (2011). The behaviour change wheel: A new method for characterising and designing behaviour change interventions. Implement. Sci..

[41] Atkins L., Francis J., Islam R., O’Connor D., Patey A., Ivers N., Foy R., Duncan E.M., Colquhoun H., Grimshaw J.M. (2017). A guide to using the Theoretical Domains Framework of behaviour change to investigate implementation problems. Implement. Sci..

[42] Gainforth H.L., Sheals K., Atkins L., Jackson R., Michie S. (2016). Developing interventions to change recycling behaviors: A case study of applying behavioral science. Appl. Environ. Educ. Commun..

[43] Morgan E., Webb L., Carter K., Goddard N. (2018). Co-designing a device for behaviour-based energy reduction in a large organisation. Proc. ACM Hum. Comput. Interact..

[44] Yun R., Aziz A., Lasternas B., Loftness V., Scupelli P., Zhang C. (2017). The persistent effectiveness of online feedback and controls for sustainability in the workplace. Energy Effic..

[45] Bao Q., Burnell E., Hughes A.M., Yang M.C. (2019). Investigating user emotional responses to eco-feedback designs. J. Mech. Des..

[46] Bao Q., Shaukat M.M., Elantary A., Yang M.C. Eco-feedback designs: A balance between the quantitative and the emotional. Proceedings of the ASME 2016 International Design Engineering Technical Conferences and Computers and Information in Engineering Conference. American Society of Mechanical Engineers.

[47] Lockton D., Harrison D., Stanton N.A. (2010). Modelling the User: How Design for Sustainable Behaviour Can Reveal Different Stakeholder Perspectives on Human Nature.

[48] Ajzen I. (2002). Perceived behavioral control, self-efficacy, locus of control, and the theory of planned behavior. J. Appl. Soc. Psychol..

